# Supplementary material for: Complexing the Oncolytic Adenoviruses Ad∆∆ and Ad-3∆-A20T with Cationic Nanoparticles Enhances Viral Infection and Spread in Prostate and Pancreatic Cancer Models
Source: Int J Mol Sci. 2022 Aug 10;23(16):8884. doi: 10.3390/ijms23168884 (PMC9408166; doi:10.3390/ijms23168884)
Supplement: Supplementary file 1 [file ijms-23-08884-s001.zip › ijms-1809957-supplementary.pdf]

Supplementary Figure S1

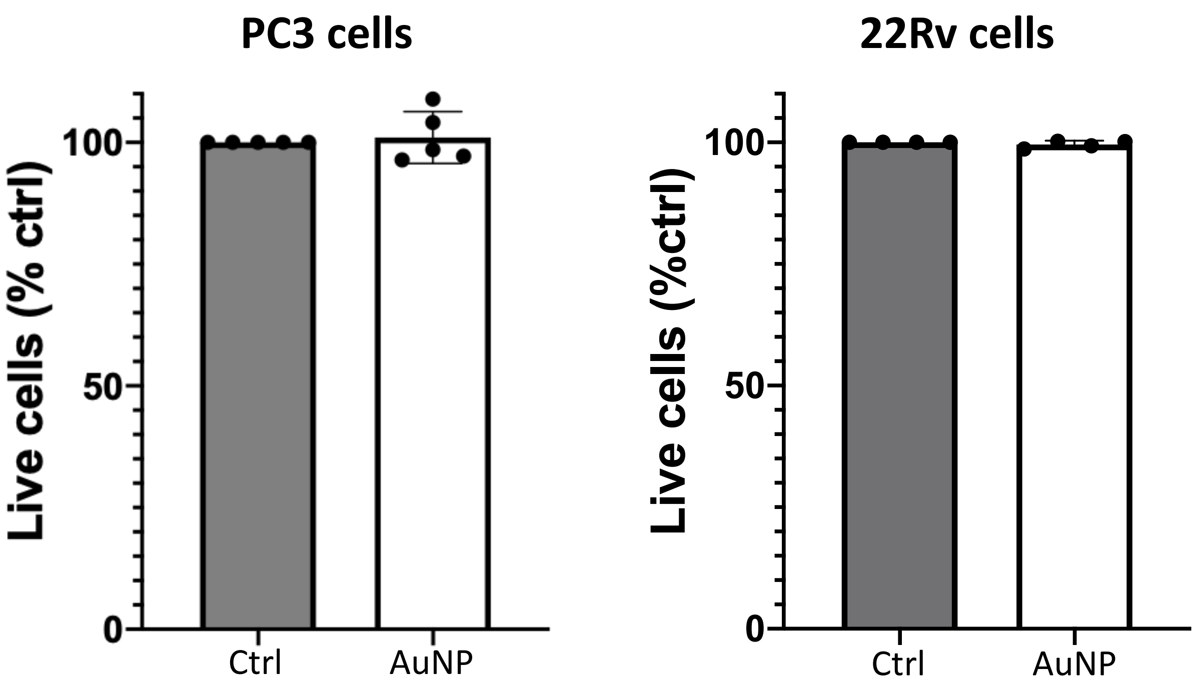

Supplementary Figure S1. AuNPs alone are not cytotoxic to PCa cells. Cells were treated with and without AuNPs at 0.5pmol. Cell viability was measured by MTS assay 4d after infection, n=4, each in triplicate.

Supplementary Figure S2

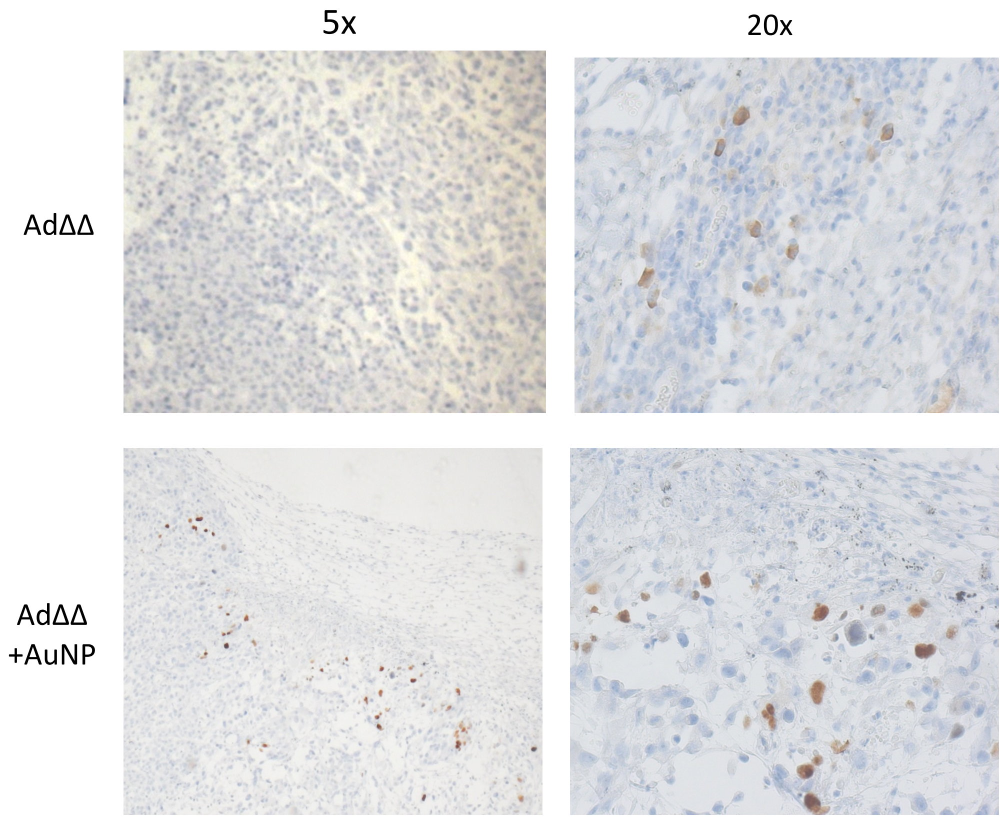

Supplementary Figure S2. Efficacious infection and spread of AdΔΔ after administration of AdΔΔ/AuNPs intravenously in PC3 xenografted tumours in athymic mice. PC3 xenografts ($5x10^6$ cells in Matrigel, 1:1), injected intravenously with AdΔΔ ($1x10^{10}$vp) ± AuNP (0.2pmol) in total 150µl. Staining for E1A and H/E 25days after viral administration, two animals/group, representative images.

Supplementary Figure S3.

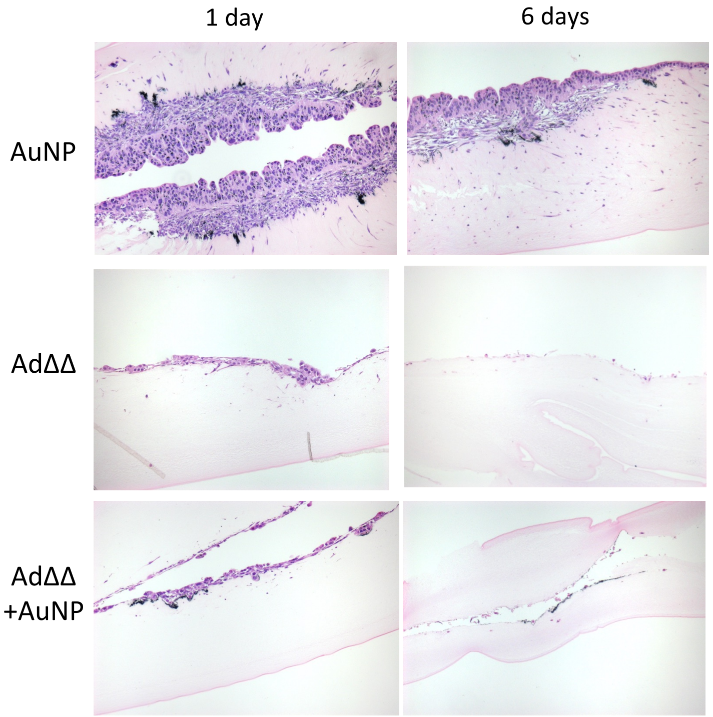

Supplementary Figure S3. AdΔΔ complexed with AuNPs efficiently infect and spread in three-dimensional co-cultures of Panc04.03:PS1 cells. Co-cultures of Panc04.03:PS1 (1:2) cells infected with AdΔΔ at 50ppc with or without AuNPs at 0.2pmol, H/E staining. Cells were cultured for 3d prior to infection with AdΔΔ ± AuNPs, and fixed and processed for IHC at 1 and 6 days of infection, 10x magnification, representative images.

Supplementary Figure S4

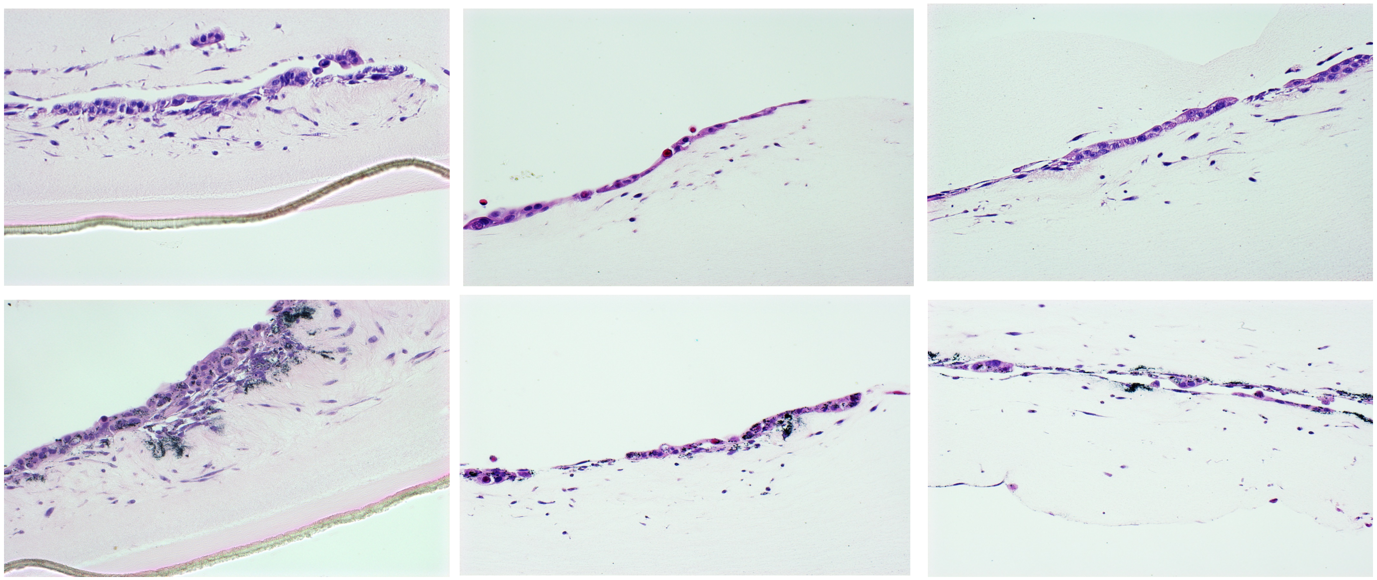

Supplementary Figure S4. Ad-3Δ-A20T complexed with AuNPs efficiently infect and spread in three-dimensional co-cultures of Panc04.03:PS1 cells. Organotypic cultures were infected with Ad-3Δ-A20T or AΔΔ at 10ppc with or without AuNPs at 0.2pmol, H/E staining after 2 days of infection, 20x magnification, representative images.
